# Practice Patterns and Barriers for Intravenous Thrombolysis: A Survey of Neurologists in Saudi Arabia

**DOI:** 10.1155/2018/1695014

**Published:** 2018-02-13

**Authors:** Mohammed H. Alanazy, Rima B. Barakeh, Alanood Asiri, Maha F. Edrees, Ahmad R. Abuzinadah, Bandar N. Aljafen, Taim Muayqil

**Affiliations:** ^1^Department of Internal Medicine, King Saud University Medical City and College of Medicine, King Saud University, Riyadh, Saudi Arabia; ^2^Department of Internal Medicine, King Abdulaziz University, Jeddah, Saudi Arabia

## Abstract

Only a small fraction of patients with acute ischemic stroke receive intravenous thrombolysis (IVT). We sought to assess barriers and practice patterns in using IVT for acute ischemic stroke among neurologists in Saudi Arabia. An electronic survey was sent to all neurologists registered with the Saudi Commission for Health Specialties. A total of 148 (77.5%) neurologists responded. The most common reported barriers for IVT administration were delayed presentation to hospitals (82.4%) and unclear time of symptom onset (50.0%). Only 9.9% of neurologists reported strict adherence to the American Heart Association/American Stroke Association guidelines for IVT administration. The most frequently waived criteria were “minor stroke with National Institutes of Health Stroke Scale [NIHSS] < 5” (49.4%) and “seizure at onset” (45.7%). For the extended 3–4.5-hour window, 18.5% of neurologists reported strict adherence to the four exclusion criteria. The most frequently waived criteria were “age older than 80 years” (53.1%) and “history of both diabetes and prior stroke” (42.0%). In conclusion, most neurologists do not adhere to the IVT exclusion criteria. However, little consensus exists regarding which criteria do not interfere with IVT administration. Barriers to IVT administration were identified and require immediate action by healthcare authorities in Saudi Arabia.

## 1. Introduction

Stroke is the second leading all-cause mortality and is a leading cause of disability worldwide [1]. In 2010, the estimated number of patients that experienced their first stroke was 16.9 million worldwide [2]. The estimated number of stroke-related deaths was 5.9 million, 71% of which occurred in low- and middle-income countries, where effective treatment may not be affordable [2]. In Saudi Arabia (SA), there are no nationwide studies on stroke incidence and prevalence, and the current literature is based on limited, older regional studies. In 1998, Al-Rajeh et al. reported an incidence rate of 29.8 per 100,000 per year for stroke in patients living in the eastern region of SA [3]. The prevalence of stroke was estimated to be 186 per 100,000 [4]. Incidence and prevalence rates of stroke in SA are relatively lower than those in western countries, which can be attributed to the predominantly young population in SA [5–7]. The American Heart Association/American Stroke Association (AHA/ASA) has developed internationally accepted guidelines for the management of acute stroke [8, 9]. However, barriers to implementation and compliance with these recommendations in SA have not been thoroughly explored. Such information may help future guideline developers to address potential regional specific limitations and take into consideration the differences in practice patterns, which may reflect cultural norms.

Intravenous thrombolysis (IVT) with recombinant tissue-type plasminogen activator (tPA) is considered the standard of care therapy for eligible patients with acute ischemic stroke if administered within 4.5 hours from symptom onset [8, 10]. When administered within this time window, IVT has been proven to improve functional outcomes. Although the IVT administration rate has markedly increased, it remains widely underused, as it is only administered to a small proportion of stroke patients (3.4–5.2%) [11]. This underuse was attributed to several reasons, including late hospital arrival, unknown time of symptom onset, and stringent eligibility criteria [12–14]. The criteria for IVT administration were drawn from the pivotal study by the National Institute of Neurological Disorders and Stroke (NINDS) that led to FDA approval for IVT within 3 hours from stroke onset [15]. However, most of the exclusion criteria were not evidence-based and were instead derived based on expert opinion at the time of the original study. Subsequent nonrandomized studies reported desirable outcomes in patients who received IVT outside of the established guidelines [16–19]. This, along with increasing experience with IVT administration and advances in neuroimaging, led to the questioning of the necessity of some of these exclusion criteria [14, 20, 21]. Recently, these criteria were modified by the FDA and AHA/ASA to allow for IVT treatment in patients that meet some of the exclusion criteria [9, 22]. However, some neurologists prefer individualized clinical judgment when evaluating patients with a relative contraindication, whereas others may strictly follow the guidelines and deny IVT.

This study sought to determine the extent to which actual practice patterns for IVT follow AHA/ASA guidelines and to assess the barriers to the use of IVT for acute ischemic stroke among neurologists in SA.

## 2. Methods

We evaluated a previously published survey (with permission from the corresponding author) [23] and modified it to include the absolute and relative IVT contraindications according to the AHA/ASA 2015 scientific statement and 2015 FDA package insert. For content validity and readability, we asked four general neurologists and one stroke neurologist to independently review the provisional version of the survey, and their inputs were incorporated into the final version (Online Supplementary Material (available here)). The survey was designed (using https://surveymonkey.com) and distributed via email and private massages during the months of June and July of 2017. We included all board-certified neurologists who are registered with the Saudi Commission for Health Specialties (SCHS), including attending neurologists (consultants including stroke neurologists and nonstroke neurologists), stroke fellows, neurology registrars, and senior registrars. Regulations for the classification of board-certified physicians are available at https://www.scfhs.org.sa. Neurology residents were excluded. To ensure maximum survey distribution, we asked a neurologist in each hospital to forward the electronic survey to other neurologists working in the same hospital. Reminder surveys were sent to nonresponders at the end of June and middle of July 2017. This study was approved by the institutional review board of King Saud University.

## 3. Statistical Analysis

Data were analyzed using SPSS statistics software version 21. Categorical data are reported as numbers and percentages. To assess whether the use of IVT in postresidency practice was dependent on IVT administration during residency, we dichotomized the responses of frequency of IVT administration to “never prescribed IVT” and “prescribed IVT as a neurologist.” Chi-square test was employed to assess whether the frequency of IVT administration as a neurologist was influenced by the experience of IVT administration during residency. Two-tailed *P* < 0.05 was considered significant.

## 4. Results

Of the 191 neurologists from 14 cities in the country, 148 (77.5%) participated and 104 (70.2%) returned a completely answered survey. Neurologists practicing in 11 of the 14 cities have administered IVT (Online Supplementary Table  1). Because of the incomplete surveys, response rate was different for each item. The majority of respondents (*n* = 99/124, 79.8%) were working in tertiary governmental hospitals, 15/124 (12.1%) were working in secondary hospitals, and 10/124 (8.1%) were working in private hospitals. Of the respondents, 86/126 (68.3%) were attending neurologists, 4/126 (3.2%) were stroke fellows, 19/126 (15.1%) were senior registrars, and 17/126 (13.5%) were registrars. More than half of the respondents (*n* = 71/125, 56.8%) were from Riyadh, and 24 (19.2%) were from Jeddah, the largest two cities in SA. The distribution of participating neurologists per city is shown in Online Supplementary Table  1.

IVT was prescribed by a significantly larger number of neurologists (*n* = 88/97, 90.7%) who had the experience of administering it during residency than by those who did not have that experience (*n* = 19/39, 48.7%), *P* < 0.001.

The frequency of IVT administration is shown in Table 1. Only 81 (60%) of neurologists prescribed IVT in the 12 months preceding the survey, and those were included in the analysis of “IVT practice patterns.” Sixty-seven of 81 (82.7%) surveys were completely answered. Criteria that would not prevent IVT administration by neurologists are shown in Table 2. Only 8/81 (9.9%) neurologists reported to adhere to the guidelines. The most frequently waived exclusion criteria were “minor stroke with National Institutes of Health Stroke Scale (NIHSS) score < 5” (*n* = 40/81, 49.4%), “seizure at onset” (*n* = 37/81, 45.7%), “ischemic stroke within 3 months” (*n* = 25/81, 30.9%), “rapidly improving stroke symptoms” (*n* = 24/81, 29.6%), and “pregnancy” (*n* = 24/81, 29.6%). All other criteria were waived by less than 25% of participating neurologists.

Regarding the extended 3–4.5-hour IVT window, 15/81 (18.5%) neurologists chose to adhere to all the four exclusion criteria (age > 80 years, history of diabetes and prior stroke, warfarin use regardless of International Normalized Ratio [INR] value, or NIHSS > 25). Of participating neurologists, 43/81 (53.1%) waived “age > 80 years,” 34/81 (42.0%) waived “history of diabetes and prior stroke,” 20/81 (24.7%) waived “NIHSS > 25,” and 17/81 (21.0%) waived “warfarin use regardless of INR value.”

Only a small percent of neurologists waived the criterion of receiving a novel oral anticoagulant (NOAC) in the last 48 hours (Table 2). The majority of neurologists (*n* = 54/81, 66.7%) reported they would not administer IVT to a patient taking dabigatran if the time of last dose was unknown, regardless of normal coagulation; 9/81 (11.1%) reported they would administer IVT if renal function is normal, and 6/81 (7.4%) reported they would administer IVT even in the setting of mild renal impairment. In patients with acute stroke while taking warfarin, 29/67 (43.3%) of neurologists reported they would use IVT with an INR of ≤1.7, and the remainder chose different INR values as shown in Table 3.

The majority of neurologists (*n* = 53/69, 76.8%) reported they would not wait for a pregnancy test result before administering IVT to a woman of childbearing age presenting with acute ischemic stroke; the remainder (*n* = 16/69, 23.2%) reported they would wait for pregnancy test results.

Less than half of the neurologists (*n* = 35/81, 43.2%) do not deny IVT based on a lower NIHSS score, but rather their decision making is based on the perceived disability of the deficit. Approximately half of the neurologists (*n* = 32/69, 46.4%) do not restrict IVT based on an upper NIHSS score, Table 3.

**Figure 1:**
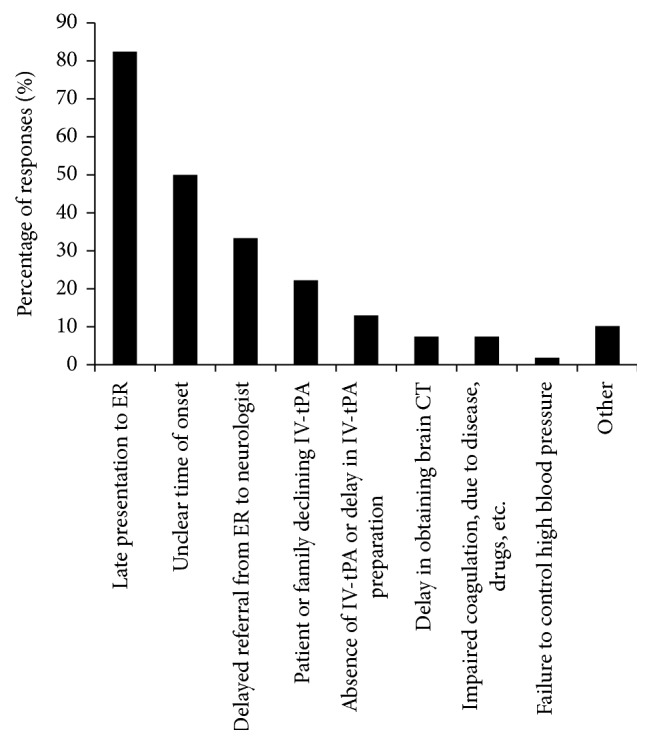
Barriers that interfere with IVT administration to patients with acute stroke as selected by neurologists (*n* = 108).

When analyzing neurologists' responses to barriers of IVT administration, we included responses from all neurologists whether or not they prescribed IVT in the past 12 months. The majority (*n* = 89/108, 82.4%) of neurologists chose “late presentation to emergency room (ER),” 54/108 (50.0%) chose “unclear time of onset,” and 36/108 (33.3%) chose “delayed referral from ER to neurologist,” Figure 1. Eleven neurologists marked “other” and provided descriptive barriers, which we classified under the following themes: (1) lack of resources including tPA, intensive care unit (ICU) bed, stroke team, trained nurses, imaging, and neuroradiologists; (2) problems with logistics including delay in triage, lack of stroke code and treatment protocol, lack of centralized stroke care, and delay in insurance approval for private hospitals; and (3) problems with consent including lack of an IVT consent and a decline or delay in signing consent by patients or their family members.

## 5. Discussion

Our study showed that there is, in general, underuse of IVT in Saudi Arabia. Similar to previous reports [12, 13], the majority of neurologists we surveyed agreed that the most common barriers interfering with IVT are late presentation to ER and unclear time of onset (82.4% and 50.0%, respectively). These results indicate lack of public awareness and are consistent with previous studies [24, 25]; thus, an initiative by the national health authorities in SA to raise public awareness of acute stroke symptoms and the time-dependent nature of treatment is warranted. Approximately one-third of neurologists we surveyed reported that in-hospital delays resulted in long door-to-needle time, emphasizing an urgent need to initiate stroke codes within emergency treatment facilities and to ensure that emergency physicians and triage nurses are trained to promptly recognize and manage potential stroke patients.

One barrier that may influence the rate of IVT use is the lack of residency training in acute stroke management. Our study showed that a higher percentage of neurologists with the experience of administering IVT during their residency subsequently prescribed IVT in their postresidency practice, as compared to those who did not have IVT experience during their residency (90.7% versus 48.7%). Although this difference in IVT use can be attributed to several factors such as infrastructural resources and logistics, our results suggest that the impact of residency training in acute stroke management plays a major role. It is not surprising that 39 (28.7%) neurologists in our cohort did not prescribe IVT during their residency since it is likely that several of them completed residency training prior to the availability of IVT for acute stroke [24]. The SCHS has taken several steps to improve neurology residency training over the past few years. Nonetheless, it is imperative to ensure that all neurology-board trainees receive sufficient training within a stroke unit to improve their decision-making skills and increase confidence when treating patients with acute stroke. There is a large unmet need of stroke care in SA. Although IVT was administered by the participating neurologists in 11 of the 14 cities, only 3 hospitals in the country, all located in Riyadh, have a dedicated stroke team and stroke unit, and few hospitals in Riyadh and Jeddah have a stroke code mostly carried out by nonstroke neurologists (personal communication, Dr. F. Al-Senani, Saudi Stroke Association). Only two centers in the country have a stroke fellowship registered with the SCHS with a total of 4 fellows. In addition, there are limited stroke neurologists in the country and, therefore, in most tertiary hospitals, IVT is administered by nonstroke neurologists. With recent advances in endovascular therapy [26, 27], it is essential to establish, in every province, comprehensive stroke centers capable of providing stroke care to patients transferred from other hospitals. Considering the limited number of neurologists and more specifically stroke neurologists in SA, the use of telestroke networks will facilitate timely IVT administration, especially in rural areas with limited resources, in which the IVT bolus dose is given in the ER, and the remaining dose infused while the patient is being transferred to a tertiary hospital (drip-and-ship) [28].

Another barrier for the underuse of IVT is the presence of one or more exclusion criteria. This study revealed that only a small percentage of neurologists (9.9%) strictly adhere to the IVT exclusion criteria (based on AHA/ASA guidelines or tPA package insert). The most commonly waived exclusion criterion was minor stroke (NIHSS < 5) (49.4%). In addition, 43.2% of neurologists reported they would not use a lower NIHSS score to withhold IVT, rather they base their decision on the perceived severity of the deficit. Conceivably, withholding IVT in patients with minor stroke (based on NIHSS) may lead to missed opportunity to treat the potentially disabling stroke symptoms with a low NIHSS (isolated aphasia, neglect, hemianopia, and gait disturbance), and symptoms not fully captured by the NIHSS, such as symptoms of posterior circulation and the right hemisphere. Previous studies report that one-third of patients denied IVT due to minor stroke or rapidly improving stroke symptoms had poor outcomes [29, 30]. The AHA/ASA guidelines recommend that patients with mild but disabling stroke within 3 hours from onset should receive IVT, as the clinical benefit has been proven [9]. Likewise, IVT should not be delayed in patients with moderate to severe stroke who demonstrate early incomplete clinical improvement to monitor further improvement. Such a delay leads to exceeding the 3-hour treatment window, especially since initial spontaneous improvement can be followed by worsening [29–31]. Approximately, one-half of neurologists in our study report they would not restrict IVT based on a higher NIHSS score. Although stroke severity is a predictor of high hemorrhage risk [32, 33], the benefit of IVT in improving functional outcomes, in otherwise eligible patients, has been proven [9]. Therefore, IVT is recommended for patients with severe acute stroke symptoms who present within 3 hours from symptom onset [9].

Seizure at stroke onset was waived by 45.7% of the neurologists we surveyed. Seizure at stroke onset was considered a relative contraindication in the AHA/ASA 2013 guidelines [8]. Additionally, according to their recent scientific statement, IVT was considered a reasonable treatment for acute stroke patients with seizure at onset, if the deficit was attributed to stroke [9]. However, seizure at stroke onset was removed from updated FDA guidelines [22].

Pregnancy is associated with an increased risk of developing acute ischemic stroke [34]. In our study, 29.6% of neurologists waived pregnancy from the exclusion criteria, and 76.8% report they would administer IVT to women of childbearing age presenting with acute stroke without waiting for a pregnancy test result. The FDA labeled tPA as a pregnancy category C [22], and pregnancy is listed as a relative contraindication in the AHA/ASA guidelines [9]. The large molecular size of tPA (7200 kDa) prevents it from crossing the placenta [35], and it is likely not teratogenic in doses used for acute stroke [9]. Since the risk of intrauterine and systemic bleeding remains a concern due to the lack of controlled studies, IVT can be used in pregnancy if the anticipated benefit of treating a moderate to severe stroke outweighs the risk of uterine bleeding [9].

Eleven (13.6%) neurologists in our cohort report they would administer IVT even if blood pressure (BP) is >185/110 mmHg. Elevated BP is associated with increased risk of symptomatic intracranial hemorrhage (sICH) [36]. The AHA/ASA statement requires that BP is lowered and stabilized below 185/110 mmHg before administering IVT, and BP must be maintained <180/105 mmHg for at least 24 hours following tPA infusion [9]. Both hyper- and hypoglycemia rarely present with a focal neurological deficit mimicking acute stroke. In our cohort, 14.8% of neurologists report they would administer IVT even with a glucose level of <50 mg/dl. It is recommended to treat high (>400 mg/dL) and low (<50 mg/dL) glucose levels first, and to administer IVT to otherwise eligible patients if the deficit persists despite optimizing glucose level [9].

In our study, a small percentage of neurologists report they would administer IVT to patients with acute stroke and an abnormal coagulation profile, or if they had received an anticoagulant within 48 hours. The thresholds of coagulation values at which the risk of bleeding outweighs the benefit of IVT have not been precisely determined. Considering the lack of robust data supporting the safety and efficacy of IVT in the presence of abnormal coagulation values (platelets < 100000/mm^3^, INR > 1.7, aPTT > 40 seconds, or PT > 15 seconds), administration of IVT is not recommended [9]. However, it is rare to have such abnormal coagulation values in stroke patients that are not previously suspected. Therefore, these laboratory results are not necessary before IVT administration, unless bleeding abnormalities, such as the concurrent use of oral anticoagulants, are suspected [9]. In our sample, only 2 neurologists (3%) report they are willing to use IVT even with an INR > 1.7 (1.8 and 1.9). Data from stroke registries suggest that IVT administration in patients on warfarin with an INR ≤ 1.7 is safe [37–39]. Oral anticoagulants are listed on the FDA warning label without a specific INR value indicated [22]. The AHA/ASA guidelines do not recommend IVT for patients on warfarin with an INR > 1.7 and/or a PT > 15 seconds, patients who received a therapeutic dose of LMWH in the last 24 hours, or patients taking a direct thrombin inhibitor or a factor Xa inhibitor, unless coagulation tests (e.g., aPTT, INR, platelet count, ecarin clotting time, thrombin time, or appropriate direct factor Xa activity assays) are normal, or more than 48 hours have passed since the last dose (assuming normal renal function) [9].

Data from the Get With The Guidelines-Stroke registry indicate that none of the European Cooperative Acute Stroke Study (ECASS) III exclusion criteria is associated with worse outcome or higher risk of sICH in the 3–4.5-hour window compared with the 0–3-hour window [40]. In our study, 18.5% of neurologists strictly adhered to the ECASS III exclusion criteria, whereas approximately 53% waived the age criteria, and 42% waived history of previous stroke and diabetes. It has previously been argued that age alone should not be a criterion to exclude elderly patients from IVT therapy [16, 40–43]. Although there was no difference in the rate of sICH between younger and older age groups (≤80 and >80 years of age), mortality rates were higher in the older age group [16, 40–43]. However, the mortality in patients >80 years was high whether or not tPA was administered [43], and this can likely be attributed to the natural history of stroke outcome in this age group [42–44]. Therefore, the AHA/ASA recommends IVT for stroke patients > 80 years of age and presenting within the 3–4.5-hour treatment window [9]. In addition, IVT is recommended for patients with prior stroke and diabetes and for patients on warfarin with an INR < 1.7, whereas the benefit of IVT for patients with NIHSS > 25 remains uncertain [9].

Our study has several limitations. We did not design our survey to assess endovascular therapy practice patterns or the use of perfusion imaging to aid in ischemic penumbra assessment. Although we had a high response rate, data were missing from 17.3% of the surveys analyzed for “IVT practice pattern,” therefore hindering the generalizability of our results. The use of a web-based survey may have introduced biases with more responses from participants having familiarity with such surveys. Considering that tPA exclusion criteria have various levels of recommendations, potential biases may have been introduced by using the phrase “bending the guideline exclusion criteria.” However, we would expect, in that case, that the majority of participants would strictly adhere to the guidelines, while in fact only a small percentage chose to adhere to the exclusion criteria listed, reflecting their knowledge of the various levels of recommendations. This is similar to a previous study in which adherence to the guidelines was reported by 18% of participants [23]. Additionally, we did not collect the neurological subspecialties of our participants, and thus we were unable to compare responses from stroke neurologists to responses from nonstroke neurologists. However, we do not think this would have changed our results as stroke care is largely provided by nonstroke neurologists in SA. The majority of our participants were from Riyadh and Jeddah, which is likely a reflection of the number of neurologists practicing in these two most populated cities of SA [45].

In conclusion, only a small percentage of neurologists report that they strictly adhere to the IVT guidelines, while the majority waived one or more exclusion criteria. There was little consensus among neurologists regarding which criteria to adhere to. Our study suggests that the AHA/ASA statement, which leaves room for clinical judgment in the presence of some relative contraindications, is more practical and well-perceived by practicing neurologists. The major barriers reported for IVT underuse in SA were delay in presentation to medical attention, unknown time of symptom onset, and in-hospital delay. These findings impose a large demand on healthcare authorities to implement measures to raise public awareness of acute stroke symptoms and signs, improve patient accessibility, transfer to stroke centers, and improve IVT administration rates for eligible stroke patients.

## Figures and Tables

**Table 1:** Frequency of intravenous tissue plasminogen activator (IV-tPA) administration.

Answers to the question “How many times have you prescribed/recommended IV-tPA for patients with acute ischemic stroke (in your postresidency practice)?”	Number of responses (%), total *N* = 136
I have never prescribed IV-tPA	29 (21.3)
I have prescribed IV-tPA in the past but it was more than 2 years ago	13 (9.6)
I have prescribed IV-tPA once in the past two years	13 (9.6)
I have prescribed IV-tPA 1–3 times in the past year	36 (26.5)
I have prescribed IV-tPA 4–6 times in the past year	15 (11.0)
I have prescribed IV-tPA 7–10 times in the past year	10 (7.4)
I have prescribed IV-tPA >10 times in the past year	20 (14.7)

**Table 2:** Number and percentage of neurologists who would administer intravenous tissue plasminogen activator (IV-tPA) in the presence of each exclusion criterion.

Criteria for the question “Which of the potential IV-tPA exclusion criteria (based on 2015 AHA/ASA scientific statement or tPA package insert) WOULD NOT necessarily prevent you from administering IV-tPA to a patient with a perceived disabling stroke (provided other inclusion/exclusion criteria are met)?” ^*∗*^Multiple answers allowed	Number of responses (%), total *N* = 81
None. I do not “bend” any of the guideline exclusion criteria	8 (9.9)
Minor stroke (NIHSS < 5)	40 (49.4)
Rapidly improving stroke symptoms	24 (29.6)
Ischemic stroke within 3 months	25 (30.9)
Significant head trauma within 3 months	9 (11.1)
Seizure at onset	37 (45.7)
Intracranial or intraspinal surgery in the prior 3 months	11 (13.6)
Blood pressure > 185/110 mm Hg despite appropriate treatment efforts to control it	11 (13.6)
Current use of anticoagulant with INR > 1.7 or PT > 15	10 (12.4)
Receiving heparin within 48 hours with aPTT value > 40 (abnormal)	6 (7.4)
Receiving direct thrombin inhibitor or factor Xa inhibitor within 48 hours with normal coagulation profile (PT, PTT, INR, platelet count, clotting time, thrombin time, factor Xa activity assays)	12 (14.8)
Receiving direct thrombin inhibitor or factor Xa inhibitor within 48 hours with unknown coagulation profile (PT, PTT, INR, platelet count, clotting time, thrombin time, factor Xa activity assays)	6 (7.4)
Receiving direct thrombin inhibitor or factor Xa inhibitor within 48 hours with abnormal coagulation profile (PT, PTT, INR, platelet count, clotting time, thrombin time, factor Xa activity assays)	6 (7.4)
Received therapeutic LMWH within 24 hours	4 (4.9)
Major surgery within 14 days	15 (18.5)
Major extracranial trauma within 14 days	12 (14.8)
Active internal bleeding	9 (11.1)
Previous ICH	15 (18.5)
Symptoms suggestive of subarachnoid hemorrhage (SAH)	6 (7.4)
Intracranial unruptured unsecured aneurysm	11 (13.6)
Intracranial remotely ruptured, secured aneurysm	13 (16.1)
Platelet count < 100,000	10 (12.4)
CT showing hypodensity > 1/3 of the cerebral hemisphere	12 (14.8)
ST elevated MI in previous 3 months	11 (13.6)
NSTEMI in previous 3 months	17 (21.0)
Gastrointestinal hemorrhage in previous 21 days	13 (16.1)
Genitourinary hemorrhage in previous 21 days	11 (13.6)
Glucose < 50 mg/dl (deficits persist after glucose correction)	12 (14.8)
Known brain tumor	6 (7.4)
Intracranial AVM (arteriovenous malformation)	9 (11.1)
Arterial puncture at a noncompressible site within 7 days	11 (13.6)
Pregnancy	24 (29.6)

**Table 3:** Number and percentage of neurologists who would administer IVT based on an INR value or NIHSS score.

Answers to the questions “In patients receiving warfarin, up to what INR do you feel comfortable administering IV-tPA?”	Number of responses (%)
≤1.4	15 (22.4)
≤1.5	11 (16.4)
≤1.6	10 (14.9)
≤1.7	29 (43.3)
≤1.8	1 (1.5)
≤1.9	1 (1.5)
≥2.0	0
*Total number*	*67*

Answers to the question “Is there an NIHSS below which you usually do not offer IV-tPA?”	Number of responses (%)

1	3 (3.7)
2	2 (2.5)
3	8 (9.9)
4	14 (17.3)
5	5 (6.2)
6	1 (1.2)
Perceived disability of the deficit	35 (43.2)
Other (please specify)	1 (1.2)
*Total number*	*81*

Answers to the question “Is there an NIHSS above which you do not offer IV-tPA?”	Number of responses (%)

>25	26 (37.7)
>30	9 (13.0)
>35	1 (1.5)
>40	1 (1.5)
I do not restrict fibrinolysis based on an upper NIHSS score.	32 (46.4)
*Total number*	*69*

## Supplementary Materials

Supplementary Materials Survey: the electronic survey distributed on neurologists. Supplementary Table 1: city-wise distribution of neurologists who participated showing tPA administration per city.
