# Supplementary material for: Practice Patterns and Barriers for Intravenous Thrombolysis: A Survey of Neurologists in Saudi Arabia
Source: Neurol Res Int. 2018 Feb 13;2018:1695014. doi: 10.1155/2018/1695014 (PMC5831958; doi:10.1155/2018/1695014)
Supplement: Supplementary Materials — Survey: the electronic survey distributed on neurologists. Supplementary Table 1: city-wise distribution of neurologists who participated showing tPA administration per city. [file 1695014.f1.pdf]

1- Survey

1. During your residency or fellowship training, Did you ever prescribe IV-tPA?

- Yes
- No

2. How many times have you prescribed/recommended IV-tPA for patients with acute ischemic stroke (in your post-residency practice)?

- I have never prescribed IV-tPA
- I have prescribed IV-tPA in the past > 2 years ago
- I have prescribed IV-tPA once in the past two years
- I have prescribed IV-tPA 1-3 times in the past year
- I have prescribed IV-tPA 4-6 times in the past year
- I have prescribed IV-tPA 7-10 times in the past year
- I have prescribed IV-tPA >10 times in the past year

3. In which city do you work?

- Riyadh
- Jeddah
- Dammam
- Madinah
- Makkah
- Al – Khubar
- Al – Ahsaa
- Al – Taif
- Abha
- Gizan
- Qassim
- Al – Baha
- Tabouk
- Al – Dahran
- Al - Qateef
- Al – Jubail
- Bisha
- Other (please specify)

4. Where do you practice?

- University hospitals
- National guard hospitals
- Military hospitals
- Ministry of health tertiary hospitals
- Ministry of health secondary hospitals
- Self-operating hospitals belonging to ministry of health
- Private sector
- Rural center
- King Faisal Specialist Hospital & Research Center
- Armed Forces Hospitals
- Other (please specify)

5.What is your job title?

- Neurology consultant
- Pediatric neurologist
- Stroke fellow
- Neurology registrar
- Neurology senior registrar

6. Which of the potential IV-tPA exclusion criteria (based on 2015 AHA guideline or tPA package insert) **WOULD NOT** necessarily prevent you from administering IV-tPA to a patient with a perceived disabling Stroke (provided other inclusion/exclusion criteria are met)? *multiple answers allowed.

- None. I don't "bend" any of the guideline exclusion criteria
- Minor stroke (NIHSS<5)
- Rapidly improving stroke symptoms
- Ischemic stroke within 3 months
- Significant head trauma within 3 months
- Seizure at onset
- Intracranial or intraspinal surgery in previous 3 months
- Blood pressure >185/110 mmHg despite appropriate treatment efforts to control it
- Current use of anticoagulant with INR>1.7or PT>15
- Receiving heparin within 48 hours with aPTT value >40 (abnormal)
- Receiving direct thrombin inhibitor within
- 48 hours with normal coagulation profile

(PT, PTT, INR, Platelet count, clotting time, thrombin time, factor Xa activity assays)

- Receiving direct thrombin inhibitor or factor Xa inhibitor within 48 hours with unknown coagulation profile profile
- (PT, PTT, INR, Platelet count, clotting time, thrombin time, factor Xa activity assays)
- Received therapeutic LMWH within 24 hours
- Major extracranial trauma within 14 days
- Active internal bleeding
- Previous ICH
- Symptoms suggestive of subarachnoid hemorrhage (SAH)
- Intracranial unruptured unsecured aneurysm
- Intracranial remotely ruptured, secured aneurysm
- Platelet count <100'000
- CT showing hypodensity >1/3of the
- Cerebral hemisphere
- ST elevated MI in previous 3 months
- NSTEMI in previous 3 months
- Gastrointestinal hemorrhage in previous 21 days
- Genitourinary hemorrhage in previous 21 days
- Glucose < 50 mg/dl
- (deficits persist after glucose correction)
- Known brain tumor
- Intracranial AVM
- (arteriovenous malformation)
- Arterial puncture at non-compressible site within 7 days
- Pregnancy

7. What additional ECASS lll contraindication criteria **WOULD NOT** necessarily prevent you from administering IV-tPA to patient with a disabling stroke in the 3-4.5 hour treatment window (provided other inclusion/exclusion criteria are met)
*multiple answers allowed

- None. I don't "bend" any of the ECASS lll criteria.
- Age older than 80 years.
- History of both diabetes and prior stroke
- Warfarin use regardless of the INR value
- NIHSS >25

8. In your experience, what is the most common barrier that interferes with administering IV-tPA in Acute stroke setting?
*multiple answers allowed (Not more than three)

- Late presentation to ER
- Delayed referral from ER to Neurologist

- Unclear time of onset
- Patient or Family declining IV-tPA
- Impaired coagulation profile, due to disease or drugs, etc
- Failure to control high blood pressure.
- Absence of IV-tPA, or delay in IV-tPA preparation.
- Delay in obtaining CT brain
- Other (please specify)

9. Do you offer IV-tPA to patients taking dabigatran who have a disabling stroke, meet inclusion/exclusion criteria for IV-tPA, and have normal PT, PTT, INR, but have unknown time from the last dose?

- Yes, even if there are mild renal function abnormalities.
- Yes, but only if the renal function is normal.
- No.

10. In a woman of childbearing age who presents with a disabling stroke and otherwise meets IV-tPA including/excluding criteria, Do you routinely wait for pregnancy test result before administering IV-tPA?

- Yes
- No

11. In patients receiving warfarin, up to what INR do you feel comfortable administering IV-tPA?

- 1.4
- 1.5
- 1.6
- 1.7
- 1.8
- 1.9
- 2.0
- 2.5
- 3.0
- 3.5
- >3.5
- Other (please specify)

12. Is there an NIHSS below which you usually do not offer IV-tPA (or dose this depend on perceived disability of the deficit)?

- 1
- 2
- 3
- 4
- 5
- 6
- perceived disability of the deficit
- Other (please specify)

13. Is there an NIHSS above which you do not offer IV-tPA ?

- >25
- >30
- >35
- >40
- I do not restrict fibrinolysis on an upper NIHSS score.
- Other (please specify)

Supplementary Table 1. City-wise distribution of neurologists who participated showing tPA administration per city.

City	Response (%)	No. of Responses	tPA given (Yes or No)
Riyadh	56.80	71	Yes
Jeddah	19.20	24	Yes
Dammam	3.20	4	Yes
Madinah	3.20	4	Yes
Makkah	3.20	4	Yes
Al-khubar	3.20	4	Yes
Al-Ahsaa	1.60	2	**No**
Al-Taif	2.40	3	Yes
Abha	1.60	2	Yes
Gizan	1.60	2	Yes
Alkharj	0.80	1	**No**
Al-Qateef	1.60	2	**No**
Al-Jubail	0.80	1	Yes
Khamees Mesheet	0.80	1	Yes
	**Total Answered**	**125**	
	**Total Skipped**	**23**	
